# Auditory environment diversity quantified using entropy from real-world hearing aid data

**DOI:** 10.3389/fdgth.2023.1141917

**Published:** 2023-04-05

**Authors:** Erik Jorgensen, Jingjing Xu, Octav Chipara, Yu-Hsiang Wu

**Affiliations:** ^1^ Department of Communication Sciences and Disorders, University of Wisconsin-Madison, Madison, WI, United States; ^2^ Starkey, Eden Prairie, MN, United States; ^3^ Department of Computer Science, University of Iowa, Iowa City, IA, United States; ^4^ Department of Communication Sciences and Disorders, University of Iowa, Iowa City, IA, United States

**Keywords:** Auditory environments, soundscape, hearing aids, entropy, ecological momentary assessment, smart health

## Abstract

**Introduction:**

Using data collected from hearing aid users’ own hearing aids could improve the customization of hearing aid processing for different users based on the auditory environments they encounter in daily life. Prior studies characterizing hearing aid users’ auditory environments have focused on mean sound pressure levels and proportions of environments based on classifications. In this study, we extend these approaches by introducing entropy to quantify the diversity of auditory environments hearing aid users encounter.

**Materials and Methods:**

Participants from 4 groups (younger listeners with normal hearing and older listeners with hearing loss from an urban or rural area) wore research hearing aids and completed ecological momentary assessments on a smartphone for 1 week. The smartphone was programmed to sample the processing state (input sound pressure level and environment classification) of the hearing aids every 10 min and deliver an ecological momentary assessment every 40 min. Entropy values for sound pressure levels, environment classifications, and ecological momentary assessment responses were calculated for each participant to quantify the diversity of auditory environments encountered over the course of the week. Entropy values between groups were compared. Group differences in entropy were compared to prior work reporting differences in mean sound pressure levels and proportions of environment classifications. Group differences in entropy measured objectively from the hearing aid data were also compared to differences in entropy measured from the self-report ecological momentary assessment data.

**Results:**

Auditory environment diversity, quantified using entropy from the hearing aid data, was significantly higher for younger listeners than older listeners. Entropy measured using ecological momentary assessment was also significantly higher for younger listeners than older listeners.

**Discussion:**

Using entropy, we show that younger listeners experience a greater diversity of auditory environments than older listeners. Alignment of group entropy differences with differences in sound pressure levels and hearing aid feature activation previously reported, along with alignment with ecological momentary response entropy, suggests that entropy is a valid and useful metric. We conclude that entropy is a simple and intuitive way to measure auditory environment diversity using hearing aid data.

## Introduction

Recent years have seen a growing interest in understanding the soundscapes or auditory environments that listeners, and specifically hearing aid users, encounter in daily life (1). Understanding the auditory environments hearing aid users encounter in daily life, and the factors that affect what types of environments hearing aid users encounter, could improve hearing aid outcomes. Hearing aid selection, hearing aid signal processing, counseling, and aural rehabilitation can be tailored based on the auditory environments users encounter and their unique hearing needs in specific environments (2–5). For example, hearing aid users with more active lifestyles, and thus who likely encounter more diverse auditory environments, may benefit more from advanced hearing aid technologies than listeners with less active lifestyles (6). Technological improvements have enabled new methods for collecting real-world environment data from daily life. One such method is to use the hearing aids to collect data about the environment, including the sound pressure level (SPL), hearing aid environment classification, and the hearing aid processing state (3,5,7). An open question, however, is how to use these data effectively to characterize the environments users encounter and draw useful conclusions. The common approach has been to describe averages and proportions, typically average sound pressure levels and proportions of environment types (3,5,7). This approach offers a limited view into the auditory environments and lifestyles of hearing aid users. Specifically, this approach does not capture the the diversity of environments or how users’ environments change over time. A key feature of modern hearing aids is their ability to adapt to environments and even, using machine learning, adapt their processing based on the diversity and types of environments an individual user encounters. Thus, it is of interest to find meaningful metrics that characterize how diverse a hearing aid user’s auditory environments are, how they change over time, and what demographic or lifestyle factors might predict these metrics.

To that end, this study presents an analysis of the data described in (7). The purpose of that study was to investigate differences in auditory environments and hearing aid feature activation encountered by different demographic groups: younger participants with normal hearing and older participants with hearing loss living in an urban or rural area. Auditory environment differences between groups were characterized using participants’ average sound pressure levels, as recorded by the hearing aids, and the proportions of different auditory environments participants encountered (speech, quiet, noise) as classified by the hearing aids. Using those metrics, the authors found that older listeners tend to encounter lower sound pressure levels than younger listeners, with the largest differences observed between the younger listeners in an urban area and the older listeners in a rural area. No differences in the proportions of environment classes was observed.

This study aims to investigate differences in auditory environment *diversity* among those groups by quantifying diversity using *entropy* of measurements collected from the participants’ hearing aids. To further validate the use of entropy as a measure of auditory environment diversity, entropy measured from hearing aid data will be compared to self-report data from ecological momentary assessments (EMA; surveys taken on a smartphone throughout the day). Entropy measures how diverse some system or parameter is as a function of its predictability. That is, the more predictable a system is—or the less often and the less drastically it changes—the less diverse it is and thus the less entropy it has (8). Entropy has been applied across a range of disciplines to quantify diversity or complexity in various ways. For example, researchers have used entropy to quantify the complexity of social networks in communities using phone call data and literature citations (9,10). Ghozi et al. (11) used entropy to show how the complexity of a single auditory environment (a college cafeteria) increased with occupancy and sound pressure level. Wu et al. (12) proposed entropy as a means to quantify auditory environments using EMA. Wu et al. (12) first showed that auditory environment diversity declined during the Covid-19 pandemic among cochlear implant users, mirroring the known effects of the pandemic on social lifestyle among the same participants (13). The authors also showed that hearing aid users with higher auditory environment entropy reported less hearing aid benefit, suggesting that entropy is a useful clinical measure for quantifying auditory environment diversity and its effect on hearing aid outcomes. The present study furthers the application of entropy to quantifying auditory environment diversity through three aims:

1. Demonstrate the use of entropy to quantify diversity of auditory environments using sound pressure level and environment classification measured by hearing aids;
2. Use entropy measured from hearing aid data to compare auditory environment diversity among younger and older listeners in an urban and rural area;
3. Compare objective differences in entropy between groups calculated from hearing aid data to self-report differences in entropy calculated from EMA.

To address these aims, entropy of SPLs and hearing aid environment classification (taken from hearing aid data) as well as entropy from EMA responses were computed for each participant. Entropy was compared among groups and data types. Methods and findings from this study can inform future investigations of auditory environment diversity and its relationship to clinical practice and audiologic outcomes.

## Materials and methods

### Participants and procedures

The dataset from (7) was used for this analysis. In that study, 46 participants were recruited in four groups: older listeners with hearing loss from an urban area (OHL-U), older listeners with hearing loss from a rural area (OHL-R), younger listeners with normal hearing from and urban area (YNH-U), and younger listeners with normal hearing from a rural area (YNH-R). The urban area was the greater San Francisco Bay Area centered around Berkeley, California, and the rural area was eastern Iowa centered around Iowa City. Urban and rural are defined in this study based on the relative population densities of the recruitment centers. The population density of Johnson County, Iowa, which contains Iowa City, is 212.9 inhabitants per square mile, while the population density of Alameda County, California, which contains Berkeley, is 2047.6 inhabitants per square mile (U.S. Census Bureau, 2019). Older was broadly defined as being over the age of 35, while younger was defined as being under the age of 35. Participants with normal hearing had to show audiometric thresholds less than 25 dB HL at all audiometric frequencies. Participants with hearing loss had to have acquired, mild-to-moderate sensorinueral hearing loss and be experienced hearing aid users. Participants in the OHL groups were generally retired, but the most participated in various volunteer, social, religious, or community groups or held part-time employment. Participants in the YNH groups were students or working professionals, and most indicated participation in a variety of additional social and community activities. Participants were paid for their time in the laboratory and $1 for each EMA they completed. Data collection took place from 2017–2019 (prior to the Covid-19 pandemic).

Participants wore Starkey Halo 2 i2400 receiver-in-the-canal hearing aids with research firmware for one week. Participants were asked to wear the hearing aids for 12–16 h per day. For participants with normal hearing, the hearing aids were set to have zero gain in all channels. For participants with hearing loss, the hearing aids were set to match the gain-frequency response of the participant’s own hearing aids using real-ear measures. For a complete description of the fitting process, see (14).

Participants also carried a smartphone connected to the hearing aids via Bluetooth. Throughout the week, data from the hearing aid was sent to and stored on the smartphone. Data included the sound level of the environment and the environment classifier as well as the processing state of the hearing aid. Due to the power limitations of the hearing aids and the smartphone, data was collected by sampling the hearing aid data, rather than collecting it continuously. Every 10 min, the system attempted to collect data for one minute at a sampling rate of 2 Hz. Every 40 min, the system attempted to collect data for 5 min at a sampling rate of 2 Hz. The purpose of the longer sampling period every 40 min was because every 40 min the smartphone delivered an EMA which asked participants to report on the auditory environment and their experience. EMAs were delivered to participants using the AudioSense+ app (15). Participants were alerted to complete a survey via a ringtone or vibration and participants could not initiate a survey themselves. For this study, only EMA questions which asked participants about the auditory environment were included in the analysis. Only EMA responses where the participant indicated that they were actively listening were included in the analysis. The EMA questions and possible responses are given in Table 1. Each question could have 1 response, with the exceptions of questions 1 and 3 which were “select all that apply.” EMA surveys were also tagged with GPS-coordinates with the consent of participants. For details on the accuracy and assessment of GPS-tagged EMA, see (2).

**Table 1:** Ecological momentary assessment (EMA) questions and answer options used in the analysis of auditory environment diversity.

Question	Response options
Q1. What did your active listening involve?	1. Conversation, live
	2. Conversation via electronic device
	3. Speech/music listening live
	4. Speech/music listening, media
	5. Environmental sound listening
Q2. (if Q1 = 1 or 2) Were you talking with more than one person?	1. Yes
	2. No
Q3. (if Q1 = 3 or 4) What kind of sounds were you listening to?	1. Speech
	2. Music
Q4. Were you in wind?	1. Yes
	2. No
Q5. Was there music in the background?	1. Yes
	2. No
Q6. Were there people around you talking in the background?	1. Yes
	2. No
Q7. How loud were the background environmental sounds?	1. Very loud
	2. Loud
	3. Medium
	4. Soft
	5. Very soft
Q8. (if Q1 = 1 or 2, or Q3 = 1) The speech of interest was ________ when compared to all other sounds.	1. Much louder
	2. Somewhat louder
	3. Equally loud
	4. Somewhat softer
	5. Much softer

Questions 1 and 3 were “select all that apply.”

### Hearing aid data

Auditory environments were quantified using two indicators, measured by the hearing aids: SPLs and environment classifier. To calculate SPL for each sampling period, all 24 channels of the hearing aids recorded the input level to that channel every 2 sec. Frequency-specific transforms were used to estimate the free-field level at each ear to account for microphone location effects. Then, microphone and pre-amp gain corrections were applied. These values were converted to dB full scale (FS) and summed. Finally, a correction factor was applied to provide an estimate of the SPL at each sample. For this study, the median value of these measurements within each sampling period for each hearing aid was calculated and the values for each hearing aid were averaged to give a single SPL value for each sampling period. The hearing aids also returned a classifier of the environment for each sample: quiet, speech, noise, music, machine, or wind. The exact nature of how the hearing aids make decisions on the environment classification is proprietary and could involve hundreds of parameters. However, the common approach is to train an algorithm using known environments and allow the algorithm to determine what acoustic features are best associated with each class and then use these features to make decisions about novel environments (4). The proportion within each sampling period the hearing aids returned each classifier was calculated and averaged between hearing aids.

### Entropy calculations

Entropy calculations were made using the Shannon entropy formulation (8):

(1) $$H(x)=-\sum_{i=1}^{n}p_{xi}\log_{10}p_{xi}$$

where $p_{xi}$ is the probability of the *i*th event in signal *x*. The use of Shannon entropy was motivated by its prior use in measuring diversity in auditory environments (11,12). Thus, for this study, entropy varies as a function of the probability of the SPLs and hearing environment classifications. One way to conceptualize the relationship between the entropy value and the SPL or classification probability is to consider the probability density function of the SPLs or hearing aid environment classification proportions for each participant. Broader, flatter probability density functions of either parameter (less predictable, more diverse) result in higher entropy values relative to narrow, peaked probability density functions (more predictable, less diverse).

SPL and environment classification entropy per subject were calculated using data from the hearing aids. SPL entropy quantifies the overall predictability or diversity of a participant’s SPLs across the entire week. The more diverse the participant’s SPLs, the less predictable the participant’s SPLs and the higher the overall entropy. SPL entropy for each participant was calculated from the time series of SPL values where each value is the median SPL in each sample across the entire week. The distributions of SPLs were discretized into 3 dB bins from 43 dB SPL to 109 dB SPL (based on the observed range of SPLs encountered by participants). The same number of bins (23), size (3 dB), and bin edges were used for each subject. To calculate entropy, the probabilities of SPLs falling into each bin were calculated ($p_{xi}$, where *x* is the time series of SPLs and *i* is the SPL bin) and multiplied by the log10 of that probability. These values were summed and multiplied by −1 to give the entropy value. Environment classification entropy quantifies the overall predictability or diversity of a participant’s environment classification across the entire week. The closer the proportions of each environment classifier, the less predictable the environment and the higher the entropy. To calculate the overall entropy for environment classification, the proportions of each environment class across the week for each participant were computed ($p_{xi}$, where *x* is the time series of environment classifications and *i* is the classifier). These values were then multiplied by the log10 of the values, summed, and multiplied by −1.

Entropy was also calculated based on participant responses on the EMA. The EMA entropy value quantifies how predictable auditory environments were for each subject based on the probabilities of pairwise combinations of EMA responses. Participants who recorded a greater diversity of EMA response combinations therefore encountered more diverse auditory environments and had higher EMA entropy values. EMA entropy was calculated using the method described in (12), which in turn used the network approach described in (9). The EMA analysis included 8 EMA questions with 52 total possible response options (Table 1). Each response option was treated as a network node. The number of links between any 2 possible nodes (EMA responses) for each subject were calculated to determine the network. From this network, entropy was calculated by determining the probability of each link between each node for each subject and computing the entropy from those values. Unlike the SPL and environment class entropy calculations, the EMA entropy calculation was dependent on the number of EMAs completed by each subject. Because entropy might increase as a function of the number of values in the calculation, the EMA entropy value was then normalized by the log of the number of EMAs completed by each subject. For a more detailed explanation of calculating entropy from EMA responses, see (12).

### Analysis

Analyses were made between all groups as well as for groups combined across age and hearing status (e.g., between YNH and OHL). Group differences were analyzed using one-way Analysis of Variance (ANOVA). When appropriate, significant omnibus statistics were followed with a priori pairwise comparisons with Tukey p-value adjustments for multiple comparisons. Model assumptions were evaluated by visually examining the data distribution and residuals, and no evidence of violating model assumptions was detected. Pearson-product moment correlation was used to assess the correlations among the 3 entropy measures. All analyses were performed in R (4.2.1, 2022-06-23, “Funny-Looking Kid”).

## Results

Hearing aid data was collected from a total of 46 participants across the 4 groups. Participants per group and summary statistics for each group are given in Table 2.

**Table 2:** Participant data.

Group	Location	N	Age (Mean, StD)	PTA (Mean, StD)
OHL-R	Iowa City, IA	13	66.2, 4.12	47.9, 6.1
OHL-U	Berkeley, CA	12	65.5, 4.12	48.3, 5.5
YNH-R	Iowa City, IA	10	25.6, 6.5	6.6, 3.7
YNH-U	Berkeley, CA	11	26.5, 4.6	2.6, 4.4

N, number of participants; Age, mean, standard deviation of age in years. PTA, mean; standard deviation of 4-frequency pure tone average (0.5 1, 2, and 4 kHz) in dB, averaged across ears.

### SPL and environment class entropy

A total of 8,292 data points from the hearing aids (SPLs and matched environment classifications) were analyzed: 1,654 for YNH-U, 2,117 for OHL-U, and 1,802 for YNH-R, 2,719 for OHL-R. The YNH showed lower numbers of data points than the OHL groups, likely because the sample size was slightly smaller for the YNH groups. However, the number of samples per participant did not differ significantly between groups (F(3)=1.68, p=0.186). Based on hearing aid data-logging information taken from the programming software, hearing aid data was collected, on average, every 20.38 min of hearing aid wear time for participants with normal hearing and every 27.1 min for participants with hearing loss. This is likely due to different amounts of daily wear time. Based on available data-logging (7 from the YNH group and 12 from the OHL group), the YNH group wore their hearings for an average of 8.71 h per day and the OHL group wore their hearing aids for an average of 13.17 h a day. This difference was significant (t(13.45)=−6.46, p<0.001). Note, however, that participants with hearing loss likely wore the hearing aids beyond data collection period each day, and thus actual data collection sampling periods were likely closer together than 27.1 min.

**Figure 1:**
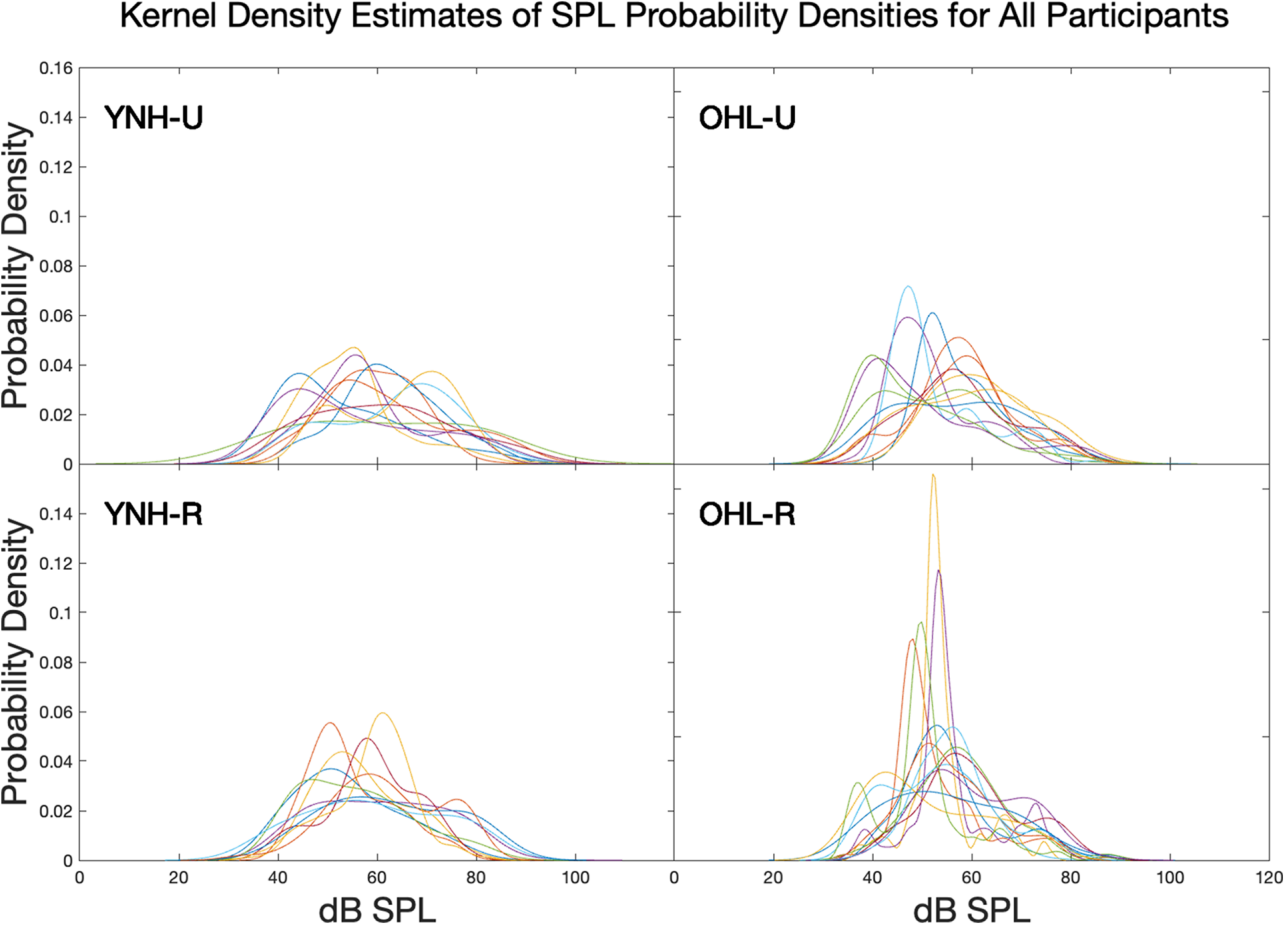
Probability density estimates for sound pressure level for each participant in each group. Broader, flatter probability densities (less predictability) result in higher entropy values.

To provide an example of how entropy quantifies diversity via predictability, kernel density estimation was used to plot the overall SPL probability density estimates for each participant (Figure 1). As can be seen, the OHL groups have generally taller, narrower probability densities than the YNH groups, indicating more predictable, less diverse auditory environments overall. Further, the rural groups generally have taller, narrower probability densities than their respective urban groups, suggesting less diversity of auditory environments in the rural groups compared to their age-matched urban groups. Taller, narrower probability densities have lower entropy values than flatter, more broad probability densities.

**Figure 2:**
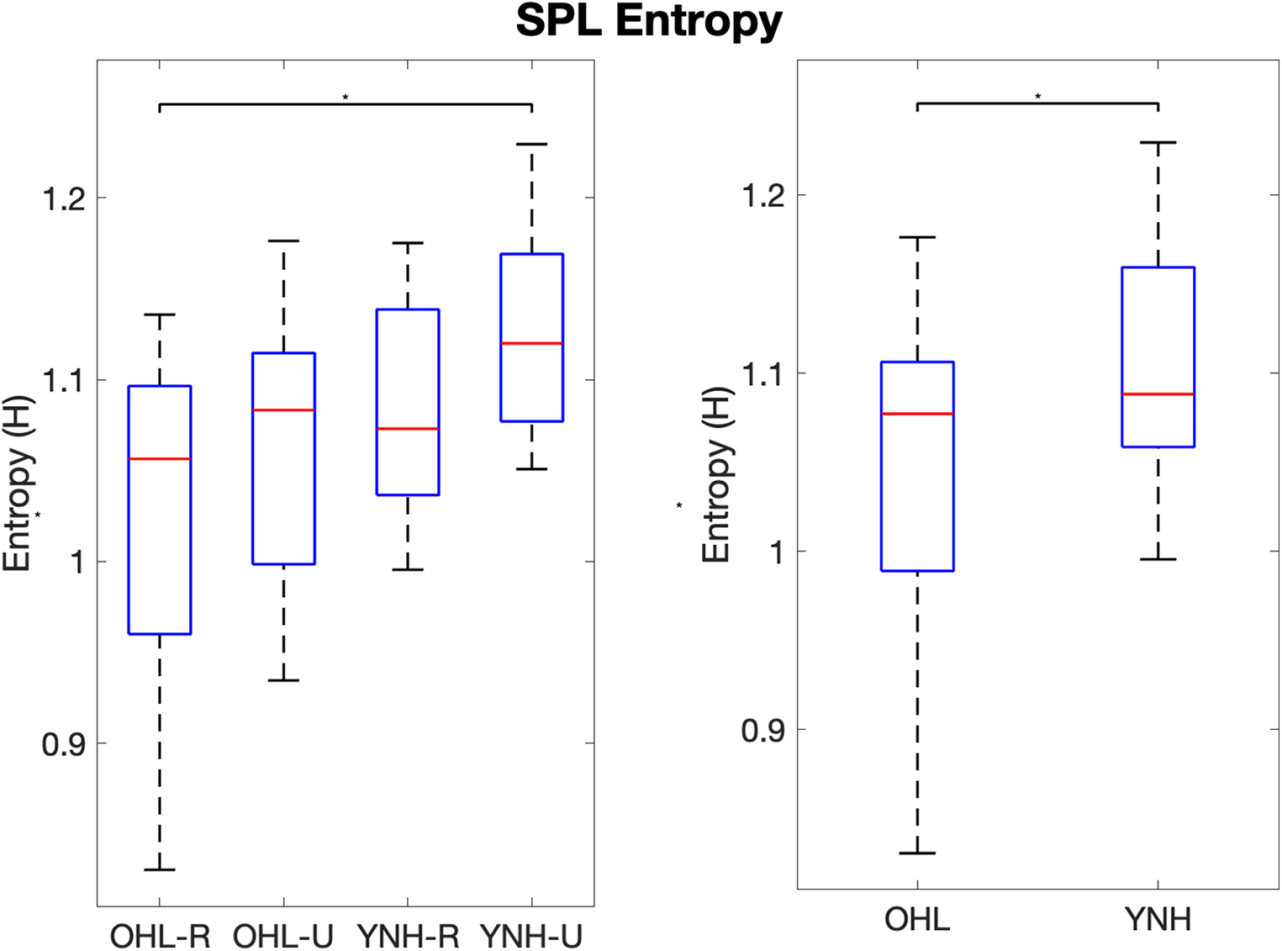
Boxplots of SPL entropy values per group (left) and groups combined along age and hearing status (right). OHL-R, older hearing loss rural; OHL-U, older hearing loss urban; YNH-R, younger normal hearing rural; YNH-U, younger normal hearing urban. OHL, older hearing loss (rural and urban combined); YNH, younger normal hearing (rural and urban combined). Vertical bars represent values within the first and third quartiles +/− the interquartile range × 1.5. Dots represent outliers. * indicates p<0.05.

This pattern is observed in the entropy values. SPL entropy values are shown in Figure 2. Generally, SPL entropy increased from the OHL groups to the YNH groups, and from the rural groups to the urban groups. There was a significant overall group effect on SPL entropy (F(3)=3.22, p=0.0321). Pairwise comparisons showed the significant omnibus statistic was driven by the YNH-U have a significantly higher SPL entropy than then the OHL-R group (t(42)=−3.01, adjusted p=0.022). Recall the taller, narrower probability density functions for the OHL groups compared to the YNH groups. This is observed in entropy values when combining the groups along age and hearing. The YNH participants had significantly higher SPL entropy than the OHL participants (F(1)=7.141, p=0.011).

**Figure 3:**
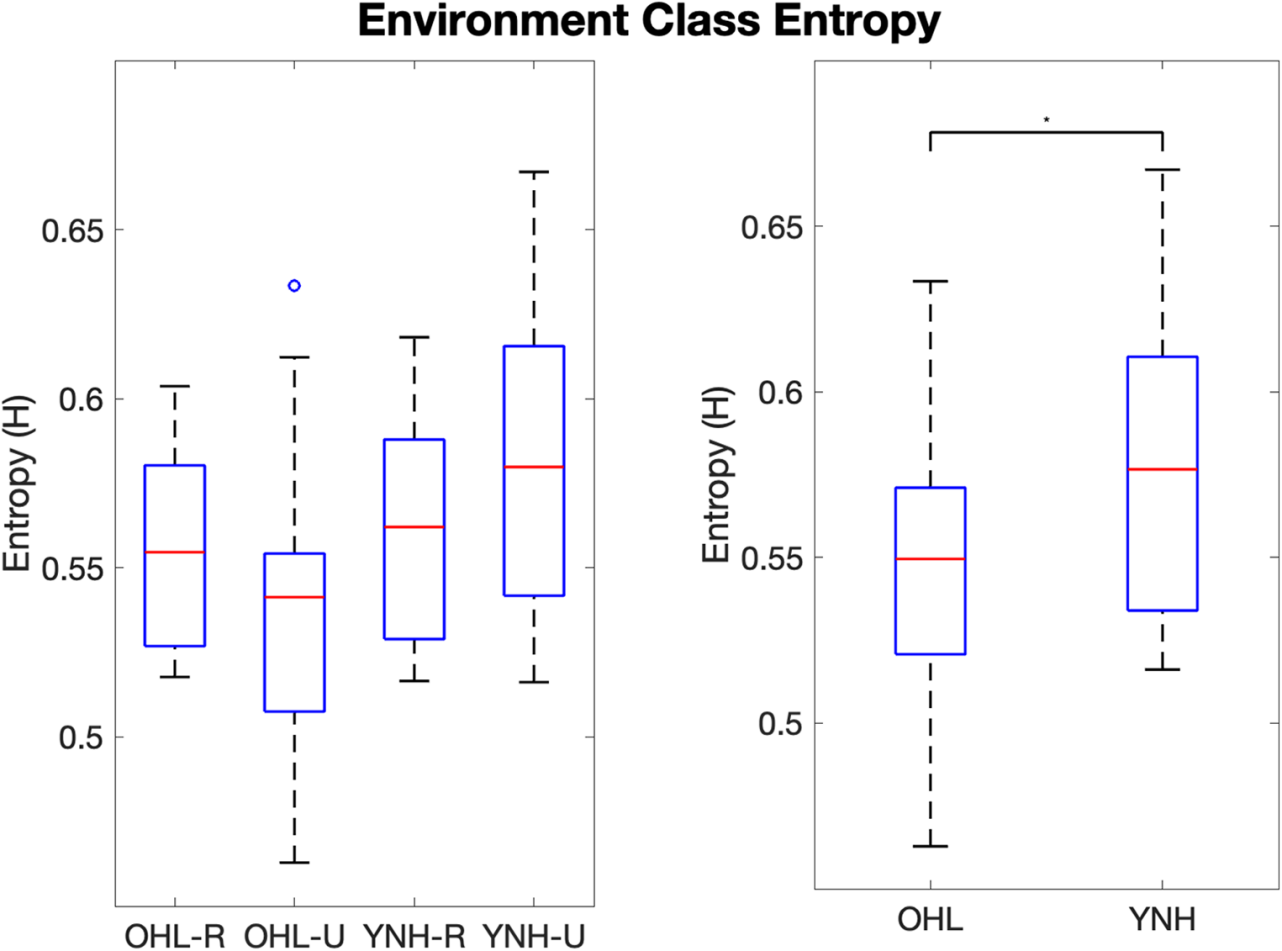
Boxplots of environment class entropy values per group (left) and groups combined along age and hearing status (right). OHL-R, older hearing loss rural; OHL-U, older hearing loss urban; YNH-R, younger normal hearing rural; YNH-U, younger normal hearing urban. OHL, older hearing loss (rural and urban combined); YNH, younger normal hearing (rural and urban combined). Vertical bars represent values within the first and third quartiles +/− the interquartile range × 1.5. Dots represent outliers. * indicates p<0.05.

Environment class entropy between all groups is shown in Figure 3. The OHL-R group had higher environment class entropy than the OHL-U group, and the YNH-U group had higher environment class entropy than the YNH-R group, though these differences did not reach significance (F(3)=2.133, p=0.11). The difference between the YNH-U and OHL-U group approached significance (t(42)=−2.492, adjusted p=0.076). When groups were combined along age and hearing status, the YNH participants had significantly higher environment class entropy than the OHL participants (F(1)=4.226, p=0.046).

### EMA entropy

2,074 ecological momentary assessments were analyzed (587 for YNH-U, 517 for OHL-U, and 286 for YNH-R, 684 for OHL-R). On average, each participant completed 8.3 EMA surveys per day (7.8 for YNH-U, 8.9 for OHL-U, 5.6 for YNH-R, and 9.9 for OHL-R). Number of EMA surveys completed per group did not differ with the exception that the YNH-R group completed fewer EMA surveys than the OHL-R group (t(42)=2.832, p=0.034).

**Figure 4:**
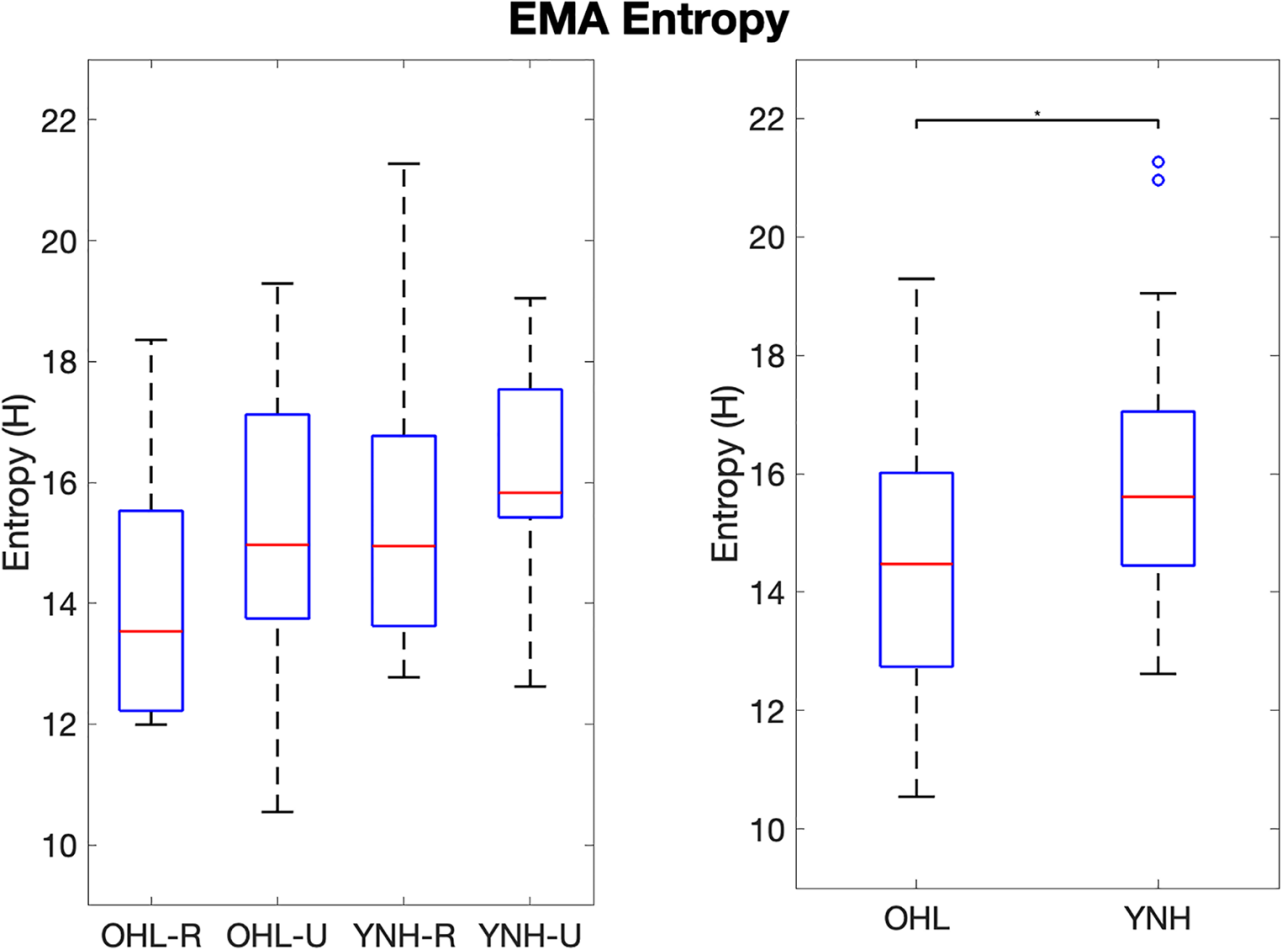
Boxplots of EMA entropy values per group (left) and groups combined along age and hearing status (right). OHL-R, older hearing loss rural; OHL-U, older hearing loss urban; YNH-R, younger normal hearing rural; YNH-U, younger normal hearing urban. OHL, older hearing loss (rural and urban combined); YNH, younger normal hearing (rural and urban combined). Vertical bars represent values within the first and third quartiles +/− the interquartile range × 1.5. Dots represent outliers. * indicates p<0.05.

EMA entropy differences between all groups followed a similar pattern as SPL entropy (Figure 4). EMA entropy was highest for participants in the YNH-U group and lowest for the OHL-R group. Differences in EMA entropy between all groups approached but did not reach significance (F(3)=1.91, p=0.15). When combined along age and hearing status, EMA entropy differences were aligned with entropy differences observed in SPL and environment class: the YNH participants had significantly higher EMA entropy than the OHL participants (F(1)=4.639, p=0.039).

### Entropy correlations

Pearson’s product-moment correlations were used to assess the relationship among the 3 entropy values. Correlation coefficients and 95% confidence interval estimates are shown in Table 3. Moderate and significant correlations were observed between SPL and environment class entropy (p=0.012) and between SPL and EMA entropy (p=0.016). The correlation between environment class and EMA entropy was not significant (p=0.265).

**Table 3:** Correlation coefficients between SPL entropy, environment class entropy, and EMA entropy.

	SPL entropy	Class entropy	EMA entropy
SPL entropy	1.00	**0.37 [0.09, 0.59]**	**0.35 [0.07, 0.58]**
Class entropy	**0.37 [0.09, 0.59]**	1.00	0.17 [−0.13, 0.44]
EMA entropy	**0.35 [0.07, 0.58]**	0.17 [−0.13, 0.44]	1.00

Values are shown with 95% confidence interval estimates. Significant correlations (p<0.05) are in bold.

## Discussion

This study aimed to use a novel metric—entropy—to quantify auditory environment diversity using SPL and environment classification data from hearing aids. We showed that entropy could be calculated in a straightforward manner from hearing aid data. The study then aimed to validate the use of entropy, calculated from hearing aid data, as a measure of auditory environment diversity by comparing SPL and environment class entropy between younger and older listeners from an urban and rural area and comparing these entropy differences to differences in entropy on EMA. SPL and environment class entropy was significantly higher for the YNH than the OHL participants, with the largest differences were observed between the YNH-U and OHL-R groups. Similarly, the YNH participants had significantly higher EMA entropy than the OHL participants. Finally, this study aimed to compare entropy measured objectively from hearing aid data to entropy measured from EMA, a self-report measure. Significant, moderate correlations were observed between SPL and environment class entropy and between SPL and EMA entropy, providing further evidence for the validity of entropy as a measure of auditory environment diversity. Taken together, the findings from this study suggest that younger listeners encounter a greater diversity of auditory environments than older listeners, that this diversity can be captured using hearing aid data and measured using entropy, and that entropy calculated using objective hearing aid data broadly corresponds with entropy measured from self-report EMA data.

Findings in the present study are broadly consistent with the findings in (7), where younger listeners were observed to encounter higher SPLs and greater likelihood of hearing aid feature activation than older listeners. This study extends those findings to formally quantify that younger listeners, and particularly younger listeners in an urban area, also encounter more diverse auditory environments than older listeners, and particularly older listeners in a rural area. In both studies, differences between the YNH and OHL groups were clear, while less clear differences were observed when groups were further divided along geographic location. A possible reason for this is that age is a more robust predictor of auditory environment diversity than geographic location, and with a relatively small sample, the clearest differences emerged when groups were combined along age. This is consistent with prior work (16). Findings from the present study are also consistent with those of Wu et al. (12), where entropy calculated from EMA was validated as a measure of auditory environment diversity. The convergence of these findings suggests that entropy is a valid measure of auditory environment diversity and that entropy measured from hearing aid data is consistent with listeners’ perceived experience.

Quantifying auditory environments via entropy with hearing aid data has some advantages over other approaches. Because data is collected frequently, it may be more sensitive to detecting auditory environment differences than other approaches. For example, as reported in (7), no differences between the groups in this study were found on the frequency sub-scale of the Auditory Lifestyle and Demand questionnaire, which aims to capture how often listeners encounter different environments and should then measure auditory environment diversity (17). Retrospective questionnaires may not be sensitive enough to capture auditory environment diversity. Both this study and Wu et al. (12) quantified auditory environment diversity using EMA. Although EMA is well-suited to collecting frequent samples about a listener’s experience, it still requires effort on the part of the participant, which in turn affects compliance, sampling frequency, and reactivity (18). Further, EMA may under-sample demanding environments like noisy places where listeners may be less likely to complete the assessment (19). Because hearing aids can collect data with no effort from the participant, the data may be less systematically biased and the sampling frequency can be very high. On the other hand, using hearing aid data to quantify auditory environments requires the user to wear the hearing aids consistently across environments, which may not always be the case (5). Finally, it is worth noting that an analysis of the data collected in this study was performed on the GPS coordinates collected from the participants’ smartphones. Those data were used to assess whether there were group differences in the number of different locations the participant went to over the week. No differences were found, suggesting that *auditory environment* diversity is not necessarily related to *environment* diversity. Thus, hearing aids, because they collect acoustic data specifically, provide a more sensitive measure of auditory environments that broader metrics like GPS tracking.

A simple, intuitive, and theoretically meaningful measure of auditory environment diversity such as entropy has many potential applications. Hearing aids users who encounter greater auditory environment diversity may benefit from more advanced hearing aid technologies such as a more detailed environment classification taxonomy, faster feature activation of adaptation, more user control, GPS-tagged features, feature parameters that learn from the environment and user input, or other algorithms that take into account less predictable inputs (2,5,6). Because auditory environment diversity can be meaningfully estimated with entropy measured from hearing aid input data, the hearing aids themselves could use this information to make decisions about how to adapt to the environment for a given user. For example, if the hearing aid determines that the user encounters a diversity of environments, based on some entropy metric, the hearing aid change adaptation parameters or classification specificity to better meet a given users’ needs. That is, rather than clinicians making choices about a users’ technology level needs based on patient interview or questionnaires, it could be left up to the hearing aid itself to decide how to employ available algorithms based on estimations of the users’ lifestyle using metrics such as SPL or environment class entropy.

This re-analysis had three key limitations that should be noted. First, absolute entropy values are not meaningful. What the actual entropy values are depends on the parameter and the study design. It is not possible to compare absolute entropy values across studies. Second, there is no ground truth for auditory environment diversity. We do not have a way to validate the entropy value by comparing it to the “true” auditory environment diversity of each participant. We validate entropy as a measure of auditory environment diversity by comparing entropy to other measures of diversity and by comparing various methods of measuring entropy. Third, this study relied on a relatively small sample from only two geographic areas. We did not attempt to collect data about all factors that might affect auditory environment diversity, such as cultural, socioeconomic, or other demographic factors. The bias in auditory environments of this sample relative to the population is unknown, and the effects of age and location on auditory environment entropy may be moderated by additional factors not accounted for in this study.

## Conclusions

Entropy, calculated using hearing aid data such as SPL and environment classification, is an intuitive, simple, and theoretically meaningful way to estimate the diversity of auditory environments encountered by listeners. SPL and environment class entropy are consistent with other measures of auditory environment diversity as well as entropy calculated using self-report on EMA, suggesting that entropy may be a valid measure of auditory environment diversity. Entropy could be a useful metric for a hearing aid to determine the auditory environment diversity of a user and make processing changes based on individual users’ auditory environment diversity.

## Data Availability

The raw data supporting the conclusions of this article will be made available by the authors, without undue reservation.
